# A Fast and Validated High Throughput Bar Adsorptive Microextraction (HT-BAµE) Method for the Determination of Ketamine and Norketamine in Urine Samples

**DOI:** 10.3390/molecules25061438

**Published:** 2020-03-22

**Authors:** Samir M. Ahmad, Mariana N. Oliveira, Nuno R. Neng, J.M.F. Nogueira

**Affiliations:** 1 Centro de Química Estrutural, Faculdade de Ciências, Universidade de Lisboa, Campo Grande, 1749-016 Lisboa, Portugal; samir.marcos.ahmad@gmail.com (S.M.A.); mariananetoliveira@hotmail.com (M.N.O.); 2 Departamento de Química e Bioquímica, Faculdade de Ciências, Universidade de Lisboa, Campo Grande, 1749-016 Lisboa, Portugal

**Keywords:** ketamine, norketamine, high throughput bar adsorptive microextraction, LVI-GC-MS(SIM), urine

## Abstract

We developed, optimized and validated a fast analytical cycle using high throughput bar adsorptive microextraction and microliquid desorption (HT-BAμE-μLD) for the extraction and desorption of ketamine and norketamine in up to 100 urine samples simultaneously, resulting in an assay time of only 0.45 min/sample. The identification and quantification were carried out using large volume injection-gas chromatography-mass spectrometry operating in the selected ion monitoring mode (LVI-GC-MS(SIM)). Several parameters that could influencing HT-BAµE were assayed and optimized in order to maximize the recovery yields of ketamine and norketamine from aqueous media. These included sorbent selectivity, desorption solvent and time, as well as shaking rate, microextraction time, matrix pH, ionic strength and polarity. Under optimized experimental conditions, suitable sensitivity (1.0 μg L^−1^), accuracy (85.5–112.1%), precision (≤15%) and recovery yields (84.9–105.0%) were achieved. Compared to existing methods, the herein described analytical cycle is much faster, environmentally friendly and cost-effective for the quantification of ketamine and norketamine in urine samples. To our knowledge, this is the first work that employs a high throughput based microextraction approach for the simultaneous extraction and subsequent desorption of ketamine and norketamine in up to 100 urine samples simultaneously.

## 1. Introduction

Ketamine (KET) was developed in 1962 during a search for a less problematic replacement for phencyclidine (PCP), an anesthetic that had gained notoriety for inducing hallucinations and psychosis. Due to its quick onset and short duration of action with only slight cardio-respiratory depression in comparison with other general anesthetics and the possibility of inhalation to maintain the anesthetic state, KET is a preferred drug for short-term surgical procedures in veterinary and human medicine, especially in children [1]. The main drawback of KET is its potential for causing vivid hallucinations, similar to those described for lysergic acid diethylamide (LSD) consumption [2]. As a result of this, it was initially abused by medical personnel and gradually became popular among young users at dance and rave parties [3]. In fact, the total quantity of KET seized worldwide increased from an annual average of 3 tons in the period 1998–2008 to 10 tons in the period 2009–2014 and 15 tons annually between 2015 and 2017 [4]. In humans, KET is metabolized in the liver by the microsomal cytochrome P450 system. CYP3A4 is the main enzyme responsible for KET *N*-demethylation to form norketamine (NKET). NKET is then hydroxylated, conjugated and excreted in the urine [5]. Studies of KET urinary excretion indicate that, over a 72-h period, little unchanged drug and NKET are present (2.3% and 1.6%, respectively). Most excreted compounds (80%) are conjugates of hydroxylated KET metabolites [6]. In urine collected from hospitalized children who had received KET as an anesthetic, it was detectable up to 2 days after drug administration (29–1410 µg L^−1^) and NKET was detected for up to 14 days (up to 1559 µg L^−1^) [7]. In a group of presumed recreational KET users, urine concentrations of KET and NKET were 6–7744 µg L^−1^ and 7–7986 µg L^−1^, respectively [8].

Several analytical methods have been described in the literature for the determination of KET or NKET in urine samples, mostly using high performance liquid chromatography with ultraviolet/visible detection (HPLC-UV) [9], gas chromatography (GC) coupled to either flame ionization detector (FID) [10], mass spectrometry (MS) [3,7,11], *Tandem* mass spectrometry (MS/MS) [12] or even liquid chromatography (LC) coupled to MS [7,8] or MS/MS [13,14]. Sample preparation techniques used in combination with these chromatographic or hyphenated systems include conventional liquid-liquid extraction (LLE) [8] or solid-phase extraction (SPE) [7,14,15], but also miniaturized techniques such as solid-phase microextraction (SPME) [16], stir bar sorptive extraction [9], hollow-fiber liquid-phase microextraction [3,10] or microextraction by packed sorbent (MEPS) [12]. However, the preparation and manipulation of these techniques are tiresome, and the number of possible simultaneous microextractions are very limited, especially when routine work is involved. In order to overcome some of these issues we recently introduced an alternative approach, high-throughput bar adsorptive microextraction (HT-BAμE) [17]. This new technique and apparatus have shown to be user-friendly, cost-effective and presented remarkable effectiveness as a rapid tool for the simultaneous microextraction of up to 100 samples. In the present work, we propose the use of HT-BAμE for the extraction process and subsequent microliquid desorption of up to 100 samples in combination with large volume injection-gas chromatography-mass spectrometry operating in the selected-ion monitoring acquisition mode (HT-BAµE-µLD/LVI-GC-MS(SIM)) for the determination of KET and NKET in urine matrices. To our knowledge this is the first work that reports the use of a miniaturized high throughput methodology for the analysis of the target compounds in urine matrices.

## 2. Results and Discussion

### 2.1. Optimization Procedure

In order to maximize the extraction efficiencies for KET and NKET in aqueous media, different parameters affecting HT-BAμE-μLD/LVI-GC-MS(SIM) procedure have been investigated and optimized using a univariate approach, were the best values for each parameter were chosen for the next optimization assay, in accordance to previous similar works [17,18]. The initial conditions consisted in spiking 1.0 mL of ultrapure water (pH 5.5) with 100 μL of a working mixture containing KET and NKET resulting in a final concentration of 181.8 μg L^−1^. Afterwards, the microextraction was performed for 90 min at 1000 rpm followed by μLD using 100 μL of MeOH containing 1.0 mg L^−1^ of IS under sonication (30 min, 42 +/− 2.5 kHz, 100 W). The parameters affecting the developed analytical approach were evaluated in a sequential order, starting from sorbent selectivity using 6 polymeric phases, which were chosen for their good performance for the extraction of polar to nonpolar compounds in aqueous media [17]. Next, the desorption solvent (MeOH, ACN and MeOH/ACN, 1/1, *v*/*v*; 100 μL) and time (from 5 to 60 min), as well as matrix pH (from 2.0 to 11.0), ionic strength (salt content from 0 to 20%, *w/v*) and polarity (organic modifier from 0 to 20%, *v*/*v*) were assayed. Finally, shaking speed (from 600 to 2200 rpm) and microextraction time (from 5 to 120 min) were evaluated.

**Figure 1:**
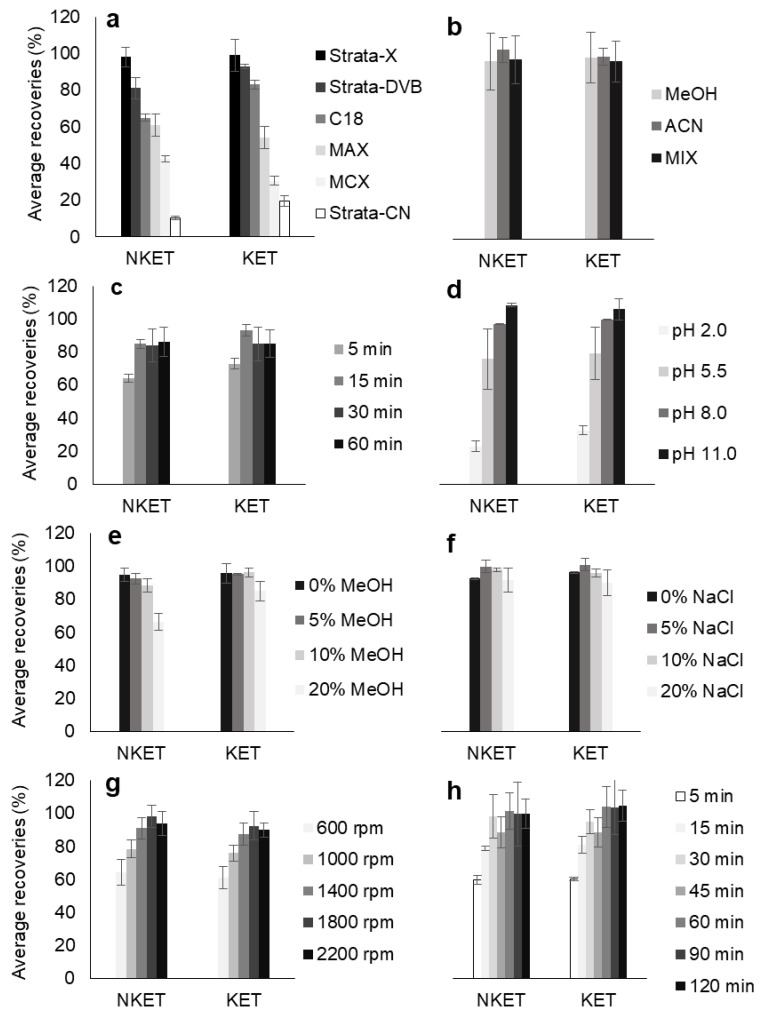
Effect of polymeric sorbent selectivity (**a**), microliquid desorption (µLD) solvent (**b**) and µLD time (**c**), matrix pH (**d**), polarity (**e**) and ionic strength (**f**), as well as shaking speed (**g**) and microextraction time (**h**) on the enrichment of ketamine (KET) and norketamine (NKET) from aqueous media, obtained by high-throughput bar adsorptive microextraction (HT-BAµE)-µLD/large volume injection-gas chromatography-mass spectrometry operating in the selected ion monitoring mode (LVI-GC-MS(SIM)). The error bars represent the standard deviation for the recovery levels of three replicates for each parameter evaluated. The microextraction devices were designed to be used only one time, once they are inexpensive and in order to avoid carryover effects [17].

Figure 1 depicts all the data from the optimization assays. The results clearly demonstrate that Strata-X presented higher recovery yields for KET and NKET than the other assayed sorbent coatings (Figure 1a). This result was expected since Strata-X promotes reverse-phase type mechanisms, such as π-π and hydrophobic interactions, which normally favors the retention of non-polar compounds through its phenyl and polystyrene groups. Moreover, this sorbent coating also allows dipole-dipole interactions, which normally favors the extraction of the more polar analytes through its pyrrolidone groups [19,20]. In general, these types of sorbents present higher selectivity for semi-polar to non-polar compounds (log P > 2.5). According to their chemical structures and polarity (Table 1), both KET and NKET present non-polar (phenyl) and polar (ketone) groups, resulting in semi-polar to non-polar characteristics (2.91 < log P < 3.35), which would favor its retention by Strata-X.

From the desorption optimization assays, it can be seen that no significant gain is achieved with either using MeOH, ACN or MeOH/ACN (1/1, *v*/*v*) to desorb KET and NKET from the NVP-DVB sorbent phase (Figure 1b). On the other hand, the optimum conditions for μLD were obtained by using 15 min of sonication time (Figure 1c).

The matrix pH, usually plays an important role in the microextraction process, where normally the non-ionized form of the target compounds seems to promote higher recovery yields from the aqueous media, since it favors the reverse-phase type interactions with the sorbent phases [17,18]. KET and NKET present weak basic characteristics (Table 1), being fully non-ionized at matrix pH > 9.5. For this reason, the recovery yields were maximized at the most alkaline pH assayed (pH 11.0)—Figure 1d.

By changing the solubility (Figure 1e) and the ionic strength (Figure 1f) of the aqueous matrix, the recovery greatly decreased for the former (especially with 20% MeOH, *v/v*) and that the recovery remained partially unchanged for the later (with successive additions of NaCl). These results can be explained by the fact that increased matrix solubility normally favors the extraction of more nonpolar or very low polarity compounds (log P > 3.5) and the increased ionic strength normally favors the extraction efficiency for compounds with high polarity (log P < 2.0) [17]. As KET and NKET present semi-polar to non-polar characteristics (2.91 < log P < 3.35), it would be expected that successive additions of NaCl or MeOH would probably hinder the microextraction process.

Finally, the effect of the shaking speed (Figure 1g) and microextraction equilibrium time (Figure 1h) for the extraction of KET and NKET in aqueous media water was also assayed. The data obtained shows that 1800 rpm is the optimum value to microextract both target analytes by using only 30 min of, with no significant improvements using higher rates.

The method development resulted in the following optimized parameters: microextraction devices coated with Strata-X sorbent phase; extraction was performed for 30 min at 1800 rpm (pH 11.0); the μLD step was performed through cavitation (42 +/− 2.5 kHz, 100 W) for 15 min using 100 μL of the MeOH containing 1.0 mg L^−1^ of IS.

### 2.2. Validation Assays

The proposed methodology was validated following the parameters in accordance to Section 3.5 which included selectivity, linearity, sensitivity, accuracy, precision, as well as recovery yields and matrix effects. Table 2 shows most of the results for the validation results. Selectivity was assessed by verifying the absence of interfering peaks in the retention times of the target compounds using blank urine samples (*n* = 10). Each calibration plot showed good linearity (*r*^2^ ≥ 0.999; residuals ≤ 9.7%) over the range of 5.0 to 1000 µg L^−1^. The linearity was also estimated (*F*_calc_) using a lack-of-fit test (at confidence interval 95%) performed for both, which was always below the *F*_tab_. As it can be observed the average recoveries yields and matrix effects using urine matrices at four spiking levels were between 84.9–105.0% (RSD ≤ 9.2%) and between −9.1–9.0% (RSD ≤ 14.1%), respectively. The accuracy values ranged from 87.2 to 110.0% (RSD ≤ 10.1%) and 85.5 to 112.1% (RSD ≤ 12.6%) for KET and NKET, respectively. These results show that the developed analytical approach is suitable for the analysis of KET and NKET in urine matrices.

### 2.3. Figures of Merit

In Table 3 we compare the LODS, linear range, accuracy, precision, recovery, sample volume and sample preparation time obtained by the proposed methodology and by other microextraction-based approaches. As it can be seen, the proposed work shows better sensitivity than most reported methodologies, even when using very sensitive instrumental systems such as GC-MS/MS [12]. The obtained LODs are only higher when compared to a methodology that uses larger amounts of sample volume [3]. The achieved accuracy, precision and recovery compares favorably with those depicted in Table 3, with the exception for a report using MEPS in combination with GC-MS/MS [12], although our proposed analytical approach uses much lower amounts of sample volume. Finally, as it can be seen, HT-BAμE-μLD/LVI-GC-MS(SIM), presents a much faster sample preparation time (0.45 min/sample) than the other microextraction-based methodologies using a high throughput configuration.

**Figure 2:**
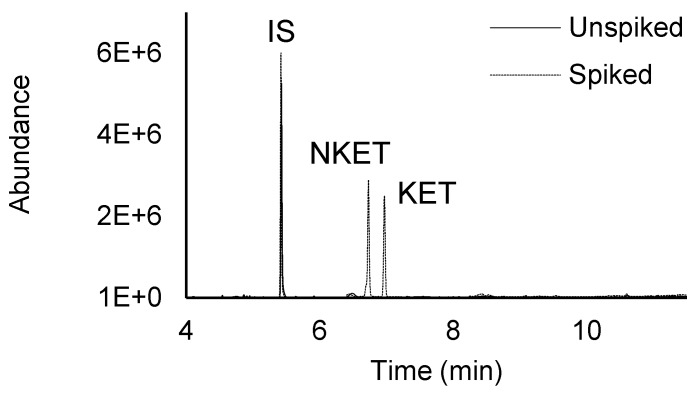
Total ion chromatogram of an assay from a spiked (125.0 μg L^−1^) and unspiked urine sample, performed by HT-BAμE-μLD/LVI-GC-MS(SIM), under optimized experimental conditions.

Figure 2 exemplifies total ion chromatograms from assays performed on spiked and unspiked urine sample, obtained by HT-BAμE-μLD/LVI-GC-MS(SIM), under optimized experimental conditions, where good selectivity and sensitivity are noticed, showing no endogenous interfering peaks at the retention times of the target compounds, including the IS.

Although the developed methodology was fully validated for the linear range of 5.0–1000.0 µg L^−1^, KET or NKT was not detected (<LOD) in the analyzed samples (*n* = 50) provided from a local clinic. However, the proposed methodology is suitable for the analysis of these compounds since urine collected from hospitalized children who had received KET as an anesthetic, it was detectable up to 2 days after drug administration (29–1410 µg L^−1^) and NKET was detected for up to 14 days (up to 1559 µg L^−1^) [7]. In groups of presumed recreational KET users, it was reported urine concentrations of KET and NKET were quantified in the range of 6–7744 µg L^−1^ and 7–7986 µg L^−1^ [8], 7.3–87.3 and 5.3–5805 µg L^−1^ [3], 5.07–23031 and 5.87–8341 µg L^−1^, respectively [14]. 

## 3. Materials and Methods

The general sample preparation approach, chemicals, reagents and sorbent materials can already be found in the literature [17]. 

### 3.1. Chemicals, Sorbents and Samples

KET hydrochloride solution (1.0 mg mL^−1^ in MeOH), (±)-NKET hydrochloride solution (1.0 mg mL^−1^ in MeOH) and diphenylamine (internal standard, IS, 98.0%) were purchased from Sigma-Aldrich (Sigma-Aldrich, MI, USA). The di-sodium hydrogen phosphate anhydrous (Na_2_HPO_4_, 99.0%) from Panreac (Barcelona, Spain). 

Stock solutions of each standard were prepared at 100.0 mg L^−1^ by proper dilution with MeOH and stored at −20 °C in amber glass flasks and renewed every month. The standard mixtures used for method development and validation were prepared by appropriate dilution of the stock solutions in MeOH. The IS stock solution was prepared at 1.0 mg L^−1^. Phosphate buffer (75.0 mmol L^−1^, pH 11.0) was prepared by proper dilution of Na_2_HPO_4_ in ultra-pure water and by adding NaOH 1.0 mol L^−1^ until the desired solution pH was established (744 pH-meter, Metrohm, Herisau, Switzerland). All the stock solutions were stored light protected at 4 °C and renewed every week. 

The authentic urine samples were provided by Joaquim Chaves Saúde clinic (Algés, Portugal). Upon arrival at the laboratory, the samples were frozen at −80 °C until use. For non-disclosure purposes, these samples were provided without any information from the donors. Blank urine samples used in all validation assays were obtained from our laboratory staff. It was specified that they could have consumed KET or any other related substances for at least a month before sampling. The study was approved by the Faculty Ethics Committee, authorization no. nr 4/2019. 

### 3.2. LVI-GC-MS(SIM) Instrumentation

The LVI-GC-MS(SIM) instrumentation specifications can also be found in a previously published manuscript [21]. In this particular case, the injection conditions were as follows: vent time, 0.49 min; flow, 50 mL min^−1^; pressure, 0 psi; purge flow, 12.9 mL min^−1^ at 2 min; the inlet temperature was programmed from 80 °C (0.5 min) to 280 °C at a rate of 600 °C min^−1^; 10 µL of injection volume at 100 µL min^−1^. The oven temperature was programmed from 80 °C (held 1 min) to 200 °C at a rate of 50 °C min^−1^, to 225 °C (held for 5 min) at a rate of 20 °C min^−1^, to 250 °C at a rate of 20 °C min^−1^ and to 280 °C at a rate of 50 °C min^−1^ resulting in 11.5 min of total running time. The solvent delay was set at 4 min. For quantification purposes, calibration curves using the internal standard methodology were performed. For method optimization in ultra-pure water, relative peak areas obtained from each assay were compared with the relative peak areas of standard controls used for spiking. In Table 1 we present the retention times (RT) and ions (*m/z*) monitored for of KET, NKET and the IS obtained by LVI-GC-MS(SIM), under optimized instrumental conditions.

### 3.3. Pre-Treatment of Urine Samples 

The urine samples were allowed to thaw and reach room temperature. The samples were vortexed for a few seconds, centrifuged for 10 min at 4500 rpm (Hermle Z 300, Germany) and the supernatants were collected. Afterwards, an acid hydrolysis was performed in order to obtain free KET or NKET from its corresponding conjugates, in accordance with the literature [15]. Therefore, 500 μL of the urine supernatants were pipetted into the microextraction vials already present in the HT-BAµE apparatus and 150 μL of HCOOH 10% (*v*/*v*) were added. Afterwards, the samples were heated to 40 °C for 1 h. After the samples were allowed to thaw and reach room temperature, 350 μL of phosphate buffer (75 mmol L^−1^, pH 11.0) and 57.5 μL of NaOH solution (10 mol L^−1^) were added in order to maintain pH 11.0. Finally, the vials were submitted to HT-BAµE-µLD analytical procedure. 

The human urine samples were collected from voluntary donors with their informed consent. 

### 3.4. HT-BAµE-µLD Methodology

After the pre-treatment step, the vials containing the samples were placed into the HT-BAµE apparatus, following a similar procedure already published but with a few alterations [17]. In the particular case, the BAµE devices were coated with NVP-DVB coating phase, the microextraction procedure was performed in an orbital shaker (Janke & Kunkel IKA-VIBRAX-VXR, Staufen, Germany) for 30 min at 1800 rpm, the microliquid desorption step was performed through cavitation (42 +/− 2.5 kHz, 100 W, Branson 3510, Carouge, Switzerland) for 15 min using 100 μL of the MeOH containing 1.0 mg L^−1^ of IS. 

### 3.5. Validation of HT-BAμE-μLD/LVI-GC-MS(SIM) Methodology

Method validation was performed in accordance to similar reported analytical approaches [13,17]. The following parameters were studied: selectivity, linearity, sensitivity, accuracy and precision, as well as recovery and matrix effects. All validation assays were performed in triplicate, except when specified otherwise.

Sensitivity was assessed through the LOD and LLOQ. The former was calculated using a signal-to-noise ratio (S/N) of 3/1. The latter was determined as the lowest concentration values that was within acceptable accuracy and precision levels, i.e., the lowest calibration level.

The calibration plots (*n* = 10) were calculated using spiked blank urine samples ranging from 5.0 to 1000.0 μg L^−1^. The linearity was estimated using lack-of-fit test, as well as by checking the respective determination coefficients (*r*^2^) and residual plots. 

Accuracy and precision were evaluated using quality control urine samples (QC) spiked with 5.0, 50.0, 200.0 and 1000.0 μg L^−1^. Inter-day precision and accuracy were evaluated in three consecutive days. Precision was expressed as the RSDs (%) of the six assays for one day and eighteen assays for three consecutive days. Accuracy followed the same procedure but calculated as relative residuals (RRs) and was expressed as percent of the nominal concentration (%). The acceptance criterion for accuracy and precision was that RRs and RSDs should be ≤ 15.0%.

Matrix effect and average recovery assays were also determined concomitantly (*n* = 6). Average recovery yields were calculated as the ratio between the mean relative peak areas of the analytes obtained from QC before microextraction and samples spiked after microextraction using four concentration levels (5.0, 50.0, 200.0 and 1000.0 μg L^−1^). Matrix effect was expressed as the ratio between the mean relative peak area obtained from QC spiked after microextraction and neat standard solutions at those same concentrations. Additionally, the RSDs of these two parameters were calculated to evaluate the variations that might arise from the matrix samples originating from different sources.

## 4. Conclusions

The methodology (HT-BAμE-µLD/LVI-GC-MS(SIM)) proposed in the present study, was fully optimized and validated to monitor KET and NKET in urine matrices. The proposed analytical cycle allowed to attain suitable analytical performance under optimized experimental conditions, including recovery, matrix effects, precision, accuracy, selectivity, sensibility and linear dynamic ranges. In addition to being user friendly, the proposed approach is environmentally friendly and cost-effective, once it takes into account the green analytical chemistry principles, i.e., uses only 100 μL of desorption solvent and 0.5 mL of urine sample per assay, does not require a derivatization step, and minimizes the overall time for the analytical procedure.

This analytical approach has the possibility of performing the microextractions and subsequent desorption of up to 100 samples in a single apparatus in just 45 min. This resulted in an average sample preparation time of 0.45 min/sample. 

To our knowledge, this is the first work that employs a high throughput based microextraction approach for the simultaneous extraction and subsequent desorption of KET and NKET in up to 100 urine samples simultaneously.

## Figures and Tables

**Table 1:** Chemical structures, octanol-water partition coefficients (log P) and acid dissociation constants (p*K*_a_), as well as retention times (RT) and ions (*m*/*z*) of KET and NKET obtained by LVI-GC-MS(SIM), under optimized instrumental conditions.

Analyte	Chemical Structure	log P ^1^	p*K*_a_^1^	RT (min)	Ions (*m/z*) ^2^
**IS**	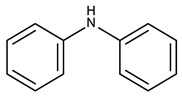	-	-	5.42	83, 168, 169
**NKET**	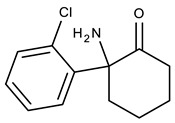	2.91	7.48	6.73	166, 168, 195
**KET**	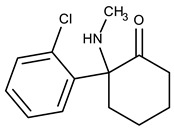	3.35	7.45	6.97	180, 182, 209

^1^ Calculator plugins were used for structure property prediction and calculation, Marvin 6.2.2, 2014, ChemAxon (http://www.chemaxon.com). ^2^ Quantification (underlined) and qualifier ions.

**Table 2:** Intraday (*n* = 6) and interday (*n* = 18) accuracy (%) and precision (± relative standard deviation (RSD), %), recovery yields (% ± RSD, %) and matrix effects (% ± RSD, %) using four spiking levels, as well as limits of detection (LODs), lower limits of quantification (LLOQs), linear ranges and *r*^2^, for KET and NKET in urine matrices, obtained by BAμE-μLD/LVI-GC-MS(SIM), under optimized experimental conditions.

Parameter	KET	NKET
**LOD (μg L^−1^)**	1.0	
**LLOQ (μg L^−1^)**	5.0	
**Linear range (μg L^−1^)**	5.0 to 1000.0	
**Calibration plot (*n* = 10)**	y = 0.0032x + 0.0066	y = 0.0032x + 0.029
***r*^2^**	0.9990	0.9970
**Intra-day assays (*n* = 6)**		
**5.0 μg L^−1^**	87.2 ± 7.6	87.5 ± 11.9
**50.0 μg L^−1^**	87.4 ± 6.6	98.8 ± 5.5
**200.0 μg L^−1^**	87.9 ± 8.5	89.0 ± 6.8
**1000.0 μg L^−1^**	94.8 ± 3.2	98.6 ± 4.5
**Inter-day assays (*n* = 18)**		
**5.0 μg L^−1^**	110.0 ± 5.7	102.0 ± 12.6
**50.0 μg L^−1^**	104.4 ± 10.1	112.1 ± 11.8
**200.0 μg L^−1^**	94.7 ± 8.7	89.8 ± 12.3
**1000.0 μg L^−1^**	102.9 ± 6.9	85.5 ± 6.1
**Recovery yields (*n* = 6)**		
**5.0 μg L^−1^**	105.0 ± 9.2	103.1 ± 5.8
**50.0 μg L^−1^**	97.8 ± 7.9	89.8 ± 4.7
**200.0 μg L^−1^**	96.6 ± 7.2	88.1 ± 8.5
**1000.0 μg L^−1^**	96.5 ± 4.0	84.9 ± 3.4
**Matrix effect (*n* = 6)**		
**5.0 μg L^−1^**	−4.4 ± 6.1	8.4 ± 6.3
**50.0 μg L^−1^**	4.9 ± 2.9	−4.6 ± 10.4
**200.0 μg L^−1^**	9.0 ± 1.5	2.5 ± 14.1
**1000.0 μg L^−1^**	−2.5 ± 6.2	−9.1 ± 5.6

**Table 3:** Comparison of the proposed method with other previously reported microextraction approaches for the determination of KET and NKET in urine samples.

Microextraction Technique	HF-LPME	MEPS	SBSE	SPME	HF-LPME	HT-BAμE
**Instrumental system**	GC-MS	GC-MS/MS	HPLC-UV	GC-MS	GC-FID	LVI-GC-MS
**LODs(μg L^−1^)**	0.1–0.25	5	2.3–9.1	100	8	1.0
**Linear range(μg L^−1^)**	0.5–50	10–250	30–3000	100–15000	3–350	5.0–1000.0
**Accuracy (%)**	88.3–108	91.4–105.6	n.a.	105.9–113.6	75.2–119.3	85.5–112.1
**Precision (%)**	≤10.1	≤9.2	≤8.9	≤14.8	≤8.9	≤12.6
**Recovery (%)**	85.2–101	72.5–100.7	90.8	n.a.	n.a.	84.9–105.0
**Sample volume (mL)**	2	0.25	3	1	3	0.5
**Sample preparation time (min/sample)**	60 ^a^	7.42 ^b^	40 ^c^	21 ^d^	20 ^c^	45
**Reference**	[3]	[12]	[9]	[16]	[10]	This work

n.a. Information not available. ^a^ Multi-tube vortexer. Number of simultaneous microextractions not available. ^b^ 8 cycles of 500 μL, 1 cycle of 250 μL and 2 cycles of 100 μL at rates of 10.0 μL s^−1^. ^c^ Magnetic stirrer. Number of simultaneous microextractions not available. ^d^ LEAP CombiPAL. Number of simultaneous microextractions not available.
